# Editorial: Metabolism and Immune Tolerance

**DOI:** 10.3389/fimmu.2018.02678

**Published:** 2018-11-15

**Authors:** Duncan Howie, Claudio Mauro

**Affiliations:** ^1^Sir William Dunn School of Pathology, University of Oxford, Oxford, United Kingdom; ^2^Institute of Inflammation and Ageing, College of Medical and Dental Sciences, University of Birmingham, Birmingham, United Kingdom

**Keywords:** immune tolerance, metabolism, regulatory T cell, transplantation, macrophage

During the past decade amongst the immunology community there has been a renaissance of interest in cellular metabolism as it relates to immune functions. Much of the work has focused on the role of metabolism in facilitating or controlling immune cell differentiation and determination of effector mechanisms. Certain fundamental relationships between metabolic state and the differentiation status of innate and lymphoid cells have been worked out, using for the most part, reductionist mouse models. One major challenge ahead will be to understand to what extent the coordination of metabolism between the multiple cell types of an immune response; lymphoid, stromal, endothelial, epithelial impacts on immunity, and immune tolerance. Our aim in assembling this Research Topic is to highlight the current understanding of cellular metabolism as it relates to immune tolerance in a variety of settings.

The collection starts with a review from Degauque et al. on the role of the inflammatory metabolic microenvironment in allogeneic transplantation. This review describes the functions of metabolites such as lactate, acetate, adenosine, and extracellular ATP on cellular and humoral immunity, and the interplay of immunosuppression and metabolism in allogeneic tolerance. Alwarawrah et al. then give an overview of the effect of nutritional status on immunity, with a focus on T cells in human and mouse. Their review highlights the effects of over and under-nutrition on the metabolism and function of the immune system in protective immunity to viruses and bacteria as well as autoimmunity. Potential therapeutic targets of glycolysis, amino acid metabolism, and mitochondrial metabolism are discussed. This is followed by primary research from Jhun et al. who address the problem of how to predict which patients who have received allogeneic liver transplants will develop tolerance over time following tapering of immunosuppressive drugs. They present data measuring circulating levels of T cell markers for Treg, Th17, Th1, and CD8 cells and correlate changes in their relative abundance with propensity to develop tolerance. The role of metabolism in the fate and function of macrophages is discussed in a comprehensive review from Diskin and Palsson-McDermott. They focus on the role of metabolic pathways, substrates, and metabolites on the programming of inflammatory or anti-inflammatory macrophages in infection and resolution of inflammation. Nguyen et al. focus on the roles of metabolism in T cells following allogeneic hematopoietic cell transplantation therapy for hematological malignancies. One undesirable consequence of this procedure is graft vs. host disease (GVHD). This review describes the potential metabolic pathways that could be targeted for therapy of GVHD and the impact of current immunosuppressive drugs on these pathways. Wawman et al. then give a thorough overview of the current state of knowledge in the metabolism of T cells in the hepatic microenvironment, a hypoxic location exposed to a rich abundance of nutrients and metabolites. They give an in-depth appraisal of the prospects for metabolic intervention for therapy in autoimmune and allo-transplant settings. The role of amino acid sensing by GCN2 and its relevance to immune regulation in metabolic and autoimmune disease is then reviewed by Battu et al. The relationship between TCR signal strength and underlying metabolic shift in human antigen-specific T cell clones is addressed in primary research by Jones et al. They demonstrate in their elegant study, using T cell clones and altered peptide ligands, that the signal strength between TCR and pHLA on antigen presenting cells governs the glycolytic shift in T cells. This observation may have therapeutic applications such as optimizing vaccination strategies. Finally, Tang and Mauro review the similarities in metabolic reprogramming between immune and endothelial cells. Immune cells and endothelial cells are intimately related in the physical space especially during diapedesis. The metabolic changes in inflammation and homeostasis in endothelial cells is important as it has huge implications for disease states such as atherosclerosis and cancer. In their review they put a spotlight on the roles of nitric oxide, hypoxia inducible factor, and adenosine monophosphate activated kinase in metabolic reprogramming of these cells.

We hope that this collection of primary research and review articles will prove useful to investigators interested in the current state-of-the-art in research into immune tolerance and cellular metabolism. We would also like to thank the many authors who generously contributed to this collection and to the Frontiers staff for their assistance.

## Author contributions

All authors listed have made a substantial, direct and intellectual contribution to the work, and approved it for publication.

### Conflict of interest statement

The authors declare that the research was conducted in the absence of any commercial or financial relationships that could be construed as a potential conflict of interest.

